# A viable but nonculturable state of Mycobacterium avium in response to macrolide antibiotics: a recipe for relapses?

**DOI:** 10.1099/jmm.0.002072

**Published:** 2025-10-30

**Authors:** Iris Schuiermanni, Eva Terschlüsen, Henrieke de Man, Jelmer Raaijmakers, Sandra Salillas, Jodie A. Schildkraut, Jakko van Ingen

**Affiliations:** 1 Department of Medical Microbiology, Radboudumc Community for Infectious Diseases, Radboud University Medical Center, PO Box 9101, 6500 HB, Nijmegen, the Netherlands; 2 Department of Medical Microbiology, Gelderse Vallei Hospital, Willy Brandtlaan 10, 6710 HN, Ede, the Netherlands; 3 Department of Microbiology, Paediatrics, Radiology and Public Health, Faculty of Medicine, University of Zaragoza, Zaragoza 50009, Spain; 4 Department of Pulmonary Diseases, Radboudumc Community for Infectious Diseases, Radboud University Medical Center, PO Box 9101, 6500 HB, Nijmegen, the Netherlands

**Keywords:** azithromycin, clarithromycin, nontuberculous mycobacteria, persister, treatment failure

## Abstract

*Mycobacterium avium* complex disease is difficult to treat, with high failure and recurrence rates despite multidrug, macrolide-based treatments. The bacterial mechanisms involved in this drug tolerance and persistence are incompletely understood. Recent evidence has suggested persistence through metabolic adaptations indicative of the viable but nonculturable state, including a decreased respiratory rate and a switch to lipid accumulation and metabolism. To assess the contribution of switching to viable but nonculturable state to macrolide tolerance, we performed time–kill kinetics assays for clarithromycin against *M. avium*. In these experiments, we performed Auramine-O (for acid-fastness, representing active mycobacteria) and Nile red (for lipid accumulation) staining and stimulation using resuscitation-promoting factors of *Mycobacterium tuberculosis*. Loss of auramine staining, increased Nile red staining and increased population sizes after stimulation with resuscitation-promoting factors support the hypothesis that clarithromycin induces a viable but nonculturable state in *M. avium*. Induction of a viable but nonculturable state is one of the mechanisms of macrolide tolerance in *M. avium*. It might be one of the drivers of the high failure and recurrence rates of macrolide-based treatments. Antimicrobials active against viable but nonculturable *M. avium* may improve treatment outcomes.

## Introduction

Macrolide antibiotics are the cornerstone of the three-drug regimen (with rifampicin and ethambutol) for the treatment of *Mycobacterium avium* complex pulmonary disease (MAC-PD) [1]. Despite prolonged multidrug treatment, recurrence rates remain high, at 30–40 % [2], suggesting that current treatment regimens do not have a sterilizing effect. Recently, it has been observed that the transcriptomic response of *M. avium* to macrolide exposure is indicative of a decreased respiratory rate [3]. We hypothesize that this results in a switch to a viable but nonculturable (VBNC) state [4] and that this is a key driver of the high recurrence rates in MAC-PD. To test this hypothesis, we performed time–kill experiments with clarithromycin against *M. avium* using resuscitation-promoting factors (RPFs) to prevent or reverse the switch to the VBNC state [4], supported by state-specific staining and microscopy.

## Methods

We collected culture filtrates containing RPFs (referred to as RPFs throughout) from mid-logarithmic phase *Mycobacterium tuberculosis* H37Rv cultures in the Mycobacterium Growth Indicator Tube system (BD Biosciences, Erembodegem, Belgium), as previously described [4]. We performed a time–kill kinetic assay in cation-adjusted Mueller–Hinton broth, as previously published [5], but with minor modifications, i.e. we exposed *M. avium* ATCC 700898 to final concentrations of 4 mg l^−1^ (i.e. 2× MIC) of clarithromycin and 4.75 ml of RPF-containing culture filtrates, i.e. in a 1 : 1 ratio. To investigate the influence of clarithromycin and RPF decay on the results, we re-exposed the bacteria to RPFs and/or clarithromycin after 7 days, by centrifugation of culture flasks, discarding the supernatant and adding fresh cation-adjusted Mueller–Hinton broth with or without RPFs and clarithromycin at 4 mg l^−1^ final concentration. We took 0.1 ml samples from all time–kill bottles on days 0, 1, 2, 3, 4, 5, 7, 8, 9, 10 and 14 for c.f.u. counting; the serially diluted samples were plated in triplicate on Middlebrook 7H10 agar plates (BD Biosciences) and incubated at 37 °C for 7 days [5]. Simultaneously, the samples were stained with Auramine-O and Nile red to determine the sizes of the auramine-positive (acid-fastness, reflecting active metabolizing state) and Nile red-positive (lipid loaded, reflecting VBNC state) mycobacterial populations [6]. Statistical analyses were done using GraphPad Prism (version 10.5.0). The response surface, i.e. area under the time–c.f.u. curve (AUC), was calculated for all conditions. To compare the effect of treatment between single conditions, we used unpaired t-tests. For the comparison of multiple treatment conditions, we performed lognormal ordinary one-way ANOVA tests of the AUCs.

## Results

**Fig. 1.**
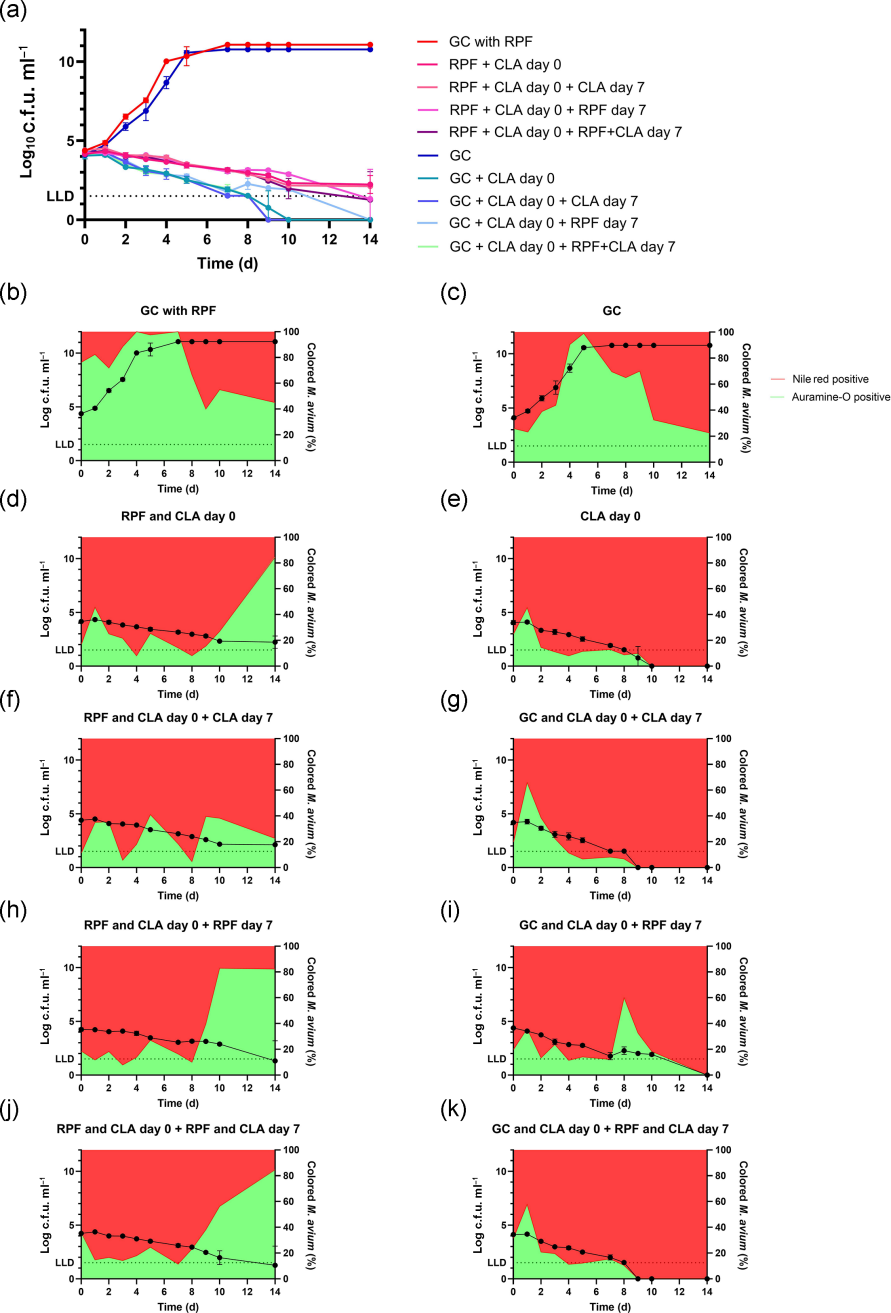
(a) Time–kill assay of *M. avium* ATCC 700898 against clarithromycin (2× MIC=4 mg l^−1^) including RPFs (in duplicate); log c.f.u. ml^−1^ plotted against time in days. RPF and clarithromycin (CLA) were added on day 0, then CLA and/or RPF were added again on day 7. Also, growth controls (GCs) were included with clarithromycin added on day 0, after which CLA and/or RPF were also added on day 7. (b–k) Dual fluorescent Auramine-O and Nile red staining on all RPF samples from time–kill assay (in duplicate); *M. avium* ATCC 700898 against clarithromycin (2× MIC=4 mg l^−1^) including RPFs. RPF and CLA were added on day 0, then CLA and/or RPF were added again on day 7. Log c.f.u. ml^−1^ of *M. avium* plotted against time in days (left *y*-axis), and percentage of stained *M. avium* plotted against time in days (right *y*-axis). Auramine-O indicates acid-fastness (actively metabolizing mycobacteria), and Nile red indicates lipid accumulation (indicative of VBNC mycobacteria). All measurements were performed in duplicate. LLD, lower limit of detection.

The addition of RPFs led to a significant acceleration of growth of *M. avium* (Fig. 1a; t=8.061, *P*=0.015), a faster conversion of the full population to an auramine stain-positive state (4 vs 5 days; Fig. 1b, c) and a slower and less re-emergence of Nile red stain-positive mycobacteria (Fig. 1b, c).

Clarithromycin steadily decreased the size of the culturable population to below the detection limit and to a complete switch to Nile red stain positivity. RPFs significantly increased the size of the culturable bacterial population throughout clarithromycin exposure (Fig. 1a; *F*=13.45, *P*=0.0318) and induced a switch to an auramine stain-positive state in the late phase of the experiment (Fig. 1d, e).

Additional clarithromycin exposure on day 7 further reduced the culturable *M. avium* population size, with a renewed shift towards a predominantly Nile red-positive population (Fig. 1f, g). The addition of RPFs on day 7 resulted in an initial stasis and then a further decrease in bacterial population size, with a shift towards auramine-positive mycobacteria (Fig. 1h, i); this shift was short-lived in the bacteria not previously exposed to RPFs (Fig. 1i), whereas it was longer lasting and more extensive in those previously exposed to RPFs (Fig. 1h). Additional exposure to both clarithromycin and RPFs on day 7 resulted in a further decrease in viable population size with an increase in auramine positivity in mycobacteria previously exposed to RPFs and clarithromycin (Fig. 1j). In mycobacteria exposed to clarithromycin but not previously exposed to RPFs, we recorded a short-lived, small expansion of the auramine-positive population, followed by a decrease in viable population size below the limit of detection and a complete switch to Nile red positivity (Fig. 1k).

## Discussion

Two lines of evidence from these experiments support the hypothesis that clarithromycin exposure drives part of the *M. avium* population into a VBNC state: (1) the increased population size of RPF-enriched *M. avium* cultures (Fig. 1a), supported by (2) the kinetics of the shift from active (auramine-positive) to VBNC (Nile red-positive) *M. avium* populations (Fig. 1b–k). These phenomena, which we were able to reproduce in the same experiment, support recent observations in transcriptomic studies that clarithromycin exposure induces respiratory – and thus likely metabolic – shutdown, i.e. a VBNC state, in *M. avium,* as well as in *Mycobacterium abscessus* [3,7]. These findings are supported by similar observations in *M. tuberculosis* [6].

The antimycobacterial activity of clarithromycin in time–kill assays and likely in other pharmacodynamic models is thus an overestimation, as the VBNC population size is not assessed. The VBNC state is not the sole antibiotic-tolerance mechanism in *M. avium*, as even after repeated RPF-induced activation, clarithromycin fails to eradicate the bacterial population; other mechanisms, including efflux pumps, also contribute [3]. The demonstration of a drug-induced VBNC state is a novelty in non-tuberculous mycobacteria (NTM) and has important implications for the treatment of MAC disease. First, macrolides are the cornerstone of treatment regimens in MAC-PD [1,2], but they drive NTM into a VBNC state [3,7] and, thus, are not sterilizing drugs. There are no antibiotics known to kill NTM in the VBNC state, with the possible exception of clofazimine, because it acts on the respiratory chain of mycobacteria [7]. Its sterilizing activity remains unproven. Second, the induction of the VBNC state by macrolides may partially explain the high number of patients who relapse rapidly after treatment [2]. Culture conversion is an important outcome measure in NTM disease [1,2], but the potential presence of VBNCs questions its true utility. It has already been observed that quantitative microbiological assays that detect actively metabolizing mycobacteria are good for predicting culture conversion, but not for predicting long-term treatment outcomes, including recurrences [8]. Following treatment discontinuation, VBNCs are likely to reactivate, inducing disease recurrence. Developing biomarkers to detect both active and VBNC mycobacteria could be useful to predict long-term treatment outcomes.

Important limitations apply. First, the metabolic state of mycobacteria in time–kill assays may not be comparable to their state during human infection, where they spend a significant amount of time intracellularly in macrophages [1,5]. Second, we use culture supernatants and not individual RPF proteins. The other components of the supernatant may also have influenced the growth of *M. avium*. Third, we use the term VBNC because it most accurately describes the phenomenon we studied, given the use of the RPF method. Yet, this descriptive term is not commonly used in mycobacteriology, where ‘persister’ and ‘non-replicating’ are more frequently used, but may not refer to the exact same phenomenon or the same bacterial population [9,10]. The VBNC state in *M. avium* and the persister state in *M. tuberculosis* may be identical bacterial cellular processes, but this has not been proven [9,10]. Also, there is limited evidence on how accurately Nile red staining reflects and detects the VBNC state. Nile red stain positivity, i.e. lipid accumulation, is an indirect effect of the switch to the VBNC state. Finally, despite increasing population sizes with RPF stimulation, we have not confirmed the actual viability of VBNC mycobacteria in the time–kill assay.

In conclusion, increased Nile red staining and a larger culturable population size upon RPF stimulation suggest that macrolides induce a VBNC state in *M. avium*. This VBNC state is likely partially responsible for the high rates of treatment failure and recurrences. To improve treatment outcomes, we need to develop truly sterilizing agents that retain activity against VBNC mycobacteria.

## Abbreviations

AUC: area under the time–c.f.u. curve
MAC-PD: *Mycobacterium avium* complex pulmonary disease
NTM: non-tuberculous mycobacteria
RPF: resuscitation-promoting factor
VBNC: viable but nonculturable
